# Type I Gaucher disease with bullous pemphigoid and Parkinson disease: A case report

**DOI:** 10.1097/MD.0000000000010188

**Published:** 2018-03-30

**Authors:** Damien Le Peillet, Virginie Prendki, Véronique Trombert, Emmanuel Laffitte, Frédéric Assal, Jean Luc Reny, Christine Serratrice

**Affiliations:** a Department of Internal Medicine, Rehabilitation and Geriatrics, University Hospital of Geneva, Trois-Chêne Hospital, Thônex; b Department of Dermatology, University Hospital of Geneva; c Department of Clinical Neurosciences, University Hospital of Geneva; d School of medicine, University of Geneva, Geneva, Switzerland.

**Keywords:** autoimmunity, bullous pemphigoid, Gaucher disease, Lewy body dementia, lysosomal disease, Parkinson disease, synuclein

## Abstract

**Rationale:**

Gaucher disease (GD) is a rare genetic lysosomal storage disorder inherited in an autosomal recessive pattern. GD is due to the deficiency of a lysosomal enzyme, acid beta-glucosidase (or glucocerebrosidase). Type 1 Gaucher disease (GD1) is characterized by thrombocytopenia, anemia, an enlarged spleen, and liver as well as bone complications (Erlenmeyer flask deformity, osteoporosis, lytic lesions, pathological and vertebral fractures, bone infarcts, and avascular necrosis leading to degenerative arthropathy). The diagnosis is usually made in first decades but is sometimes delayed. Parkinson disease, neoplasia, and immune system abnormalities may be associated with GD1.

**Patient concerns:**

A patient known for hepatosplenomegaly with hyperferritinemia, anemia, and thrombocytopenia was admitted for Lewy body dementia and bullous pemphigoid.

**Diagnoses:**

Type 1 Gaucher disease.

**Intervention:**

No specific treatment started.

**Outcomes:**

patient died ten months later due to pneumonia.

**Lessons:**

To the best of our knowledge, this is the first case of the association between GD1, bullous pemphigoid, and Lewy body dementia. We discuss the central role of alpha-synuclein in these pathologies.

## 1 Introduction

Type 1 Gaucher disease (GD1) is a rare lysosomal autosomal recessive disease with a significant and burdensome morbidity. Mutations in the glucocerebrosidase gene (*GBA1*) gene encoding this enzyme may cause Gaucher disease (GD). More than 300 mutations have been described. GD1 is characterized by hepatosplenomegaly, cytopenia, fatigue, and bone involvement.^[1]^ Delays in diagnosis can have significant physical and psychological consequences. Numerous comorbidities as Parkinson disease (PD),^[2]^ neoplasia,^[3]^ and immune system abnormalities^[4]^ may be associated with GD1.

## 2 Case report

A 62-year-old man was admitted for further evaluation for multisystem synucleinopathy diagnosed with Lewy body dementia (LBD).

The patient was diagnosed with PD at the age of 44 following of a right-hand rest tremor that responded to levodopa. After 3 years, he developed motor fluctuations and mild cognitive impairment, and 8 years after onset he presented with severe axial and bilateral Parkinsonism, dysphagia, visual hallucinations, and the clinical criteria for PD with dementia. At that time, he was bedridden and had to be fed by gastrostomy. This patient had known hepatosplenomegaly with hyperferritinemia, anemia, and thrombocytopenia since 2010. A non-alcoholic steatohepatitis was suspected. Confirmation by liver biopsy was not performed due to his general poor health. Two years ago, he presented with bullous lesions to his hands and feet. Bullous pemphigoid (BP) was confirmed by skin biopsy and positive circulating autoantibodies: anti-BP180: 27.5 UI/mL (N < 9); and anti-BP230: 35.4 UI/mL (N < 9). He was successfully treated with prednisone, tetracycline, and nicotinamide.

Personal medical history included 2 episodes of infectious and ischemic colitis, as well as a laparoscopic cholecystectomy for multiple events of cholelithiasis.

In his family history, 9 siblings had undergone a cholecystectomy, a sister had died at 50 years old with PD, and 4 siblings died at birth.

At admission, physical examination showed a hepatosplenomegaly, a mitral systolic murmur, and a severe axial and bilateral Parkinsonian syndrome. Laboratory tests showed low platelets at 99,000/mm,^[3]^ increased gamma glutamyl transferase at 2N, increased ferritin (624 μg/L), monoclonal IgA (IgA concentration: 8.37 g/L, kappa/lambda ratio: 5.07).

A computed tomography scan demonstrated a homogenous splenomegaly (bipolar diameter: 19 cm) and hepatomegaly (craniocaudal diameter: 18 cm) with diffuse osteopenia.

Given thrombocytopenia with splenomegaly, hyperferritinemia, and monoclonal gammopathy, a GD1 was suspected and confirmed by a beta glucocerebrosidase rate < 1.0 μmol/h, with lyso Gb1 at 354 ng/mL (N < 4.8 ng/mL), and chitotriosidase at 22.3 nmol/min per mL (N = 0.15–0.90). Genetic analysis found 2 *GBA1* variants: c.1226A>G (p.Asn409Ser) and c.1342G>C (pAsp448His).

Bone radiography did not reveal any specific features, but it was impossible to perform skeletal magnetic resonance imaging and X-ray absorptiometry. Echocardiography showed mild pulmonary hypertension.

The patient died 10 months later due to pneumonia.

Patient's daughter gave an oral consent for the publication.

## 3 Discussion

Numerous comorbidities have been associated with GD1, usually occurring after the disease's diagnosis. However, if the diagnosis of GD1 is delayed, these comorbidities can precede that diagnosis. The best-known comorbidities are PD, hematological and nonhematological malignancies, monoclonal gammopathies, and immune disorders.

The link between GD and PD is well established. After the first case reports from 1996,^[2,5]^ associations between *GBA1* mutations, PD, and LBD are now clear. Moreover, carriers of *GBA1* mutations might be at an increased risk of developing PD.^[6,7]^ Even though the pathophysiology is not yet completely understood, *GBA1* mutations lead to a dysfunction of the autophagy–lysosomal pathway and then to a decreased degradation of alpha-synuclein. In patients with PD and *GBA1* mutations, age at PD diagnosis is younger and dementia is usually more severe. To date, specific therapies for GD do not affect PD symptoms.

Monoclonal gammopathies, but also polyclonal gammopathies and autoimmune disorders, have been described during GD. Chronic stimulation of the immune system is a constant hallmark of GD and has relevant consequences. In a prospective study, we previously showed that autoantibodies tend to be more frequent in GD1 patients than in healthy control subjects.^[4]^ Fifty-two percent of GD1 patients had serum autoantibodies (such as anticardiolipins and antinuclear antibodies). Despite the presence of such autoantibodies, even with high titers, no patients displayed any symptoms of autoimmune disease. However, some cases of autoimmune diseases in patients with GD1 have been reported as autoimmune hemolytic anemia,^[8]^ antiphospholipid syndrome,^[9]^ lupus, and immune thrombocytopenia.^[10]^ The accumulation of lipids in GD cells is associated with an inflammatory state, an activation of macrophages, and cytokine secretion.^[11,12]^ Some of these interleukins, including interleukin-6, interleukin-1, interleukin-10, or interleukin-2 receptor, are associated with immune-system hyperactivity and related to the development of monoclonal or polyclonal gammopathies. Moreover, lysosomes play a key role in antigenic presentation through major histocompatibility complex molecules I and II, and cluster of differentiation 1 molecules. BP is a bullous autoimmune disease affecting elderly patients. The pathogenesis is in part associated with autoantibodies against skin antigens BP180 (termed collagen XVII) and BP230. Usually, BP affects people in their late 70s, and it is associated with several comorbidities.^[13]^ The association between BP and neurodegenerative pathologies was first suggested in some clinical cases and then in population-based studies and meta-analysis. Some retrospective studies have reported an association with PD.^[14]^ It is interesting to note that these autoantigens are also expressed in the central nervous system, and collagen XVII autoantibodies are present in PD.^[15]^ Although synuclein is also present in the skin the pathogenic link between BP and PD is poorly known.

In the present case, we can hypothesize that the subject's BP is either linked to PD, as previously demonstrated by clinical cases and a meta-analysis, or to GD when we consider the immune abnormalities present in this lysosomal disease. Neither anti-BP180 nor anti-BP230 antibodies have previously been described or searched for in GD1. As PD and GD are synucleinopathies, we wonder whether synuclein, which is present in the skin, has a role in BP.

## 4 Conclusion

We report the first case of GD associated with PD, LBD, and BP. BP could be linked to PD and GD and synuclein could be involved in both. This report also highlights the difficulties in diagnosing rare diseases like lysosomal diseases, especially in elderly patients, and the possible effects of delays in that diagnosis.

## Acknowledgments

The authors thank Professor Jacques Serratrice for helpful discussion and Mr Darren Hart for the English translation. Jacques Serratrice and Darren Hart gave permission to be cited.

## Author contributions

**Supervision:** C. Serratrice.

**Writing – original draft:** C. Serratrice, D. Le Peillet

**Writing – review & editing:** C. Serratrice, D. Le Peillet, E. Laffitte, F. Assal, J.L. Reny, V. Prendki, V. Trombert.
